# Cytochrome *c* biogenesis System I

**DOI:** 10.1111/j.1742-4658.2011.08376.x

**Published:** 2011-11

**Authors:** Julie M Stevens, Despoina A I Mavridou, Rebecca Hamer, Paraskevi Kritsiligkou, Alan D Goddard, Stuart J Ferguson

**Affiliations:** 1 Department of Biochemistry, University of OxfordUK; 2 Department of Statistics, University of OxfordUK

**Keywords:** Ccm, cytochrome *c*, cytochrome *c* biogenesis, System I

## Abstract

Cytochromes *c* are widespread respiratory proteins characterized by the covalent attachment of heme. The formation of *c*-type cytochromes requires, in all but a few exceptional cases, the formation of two thioether bonds between the two cysteine sulfurs in a –CXXCH– motif in the protein and the vinyl groups of heme. The vinyl groups of the heme are not particularly activated and therefore the addition reaction does not physiologically occur spontaneously in cells. There are several diverse post-translational modification systems for forming these bonds. Here, we describe the complex multiprotein cytochrome *c* maturation (Ccm) system (in *Escherichia coli* comprising the proteins CcmABCDEFGH), also called System I, that performs the heme attachment. System I is found in plant mitochondria, archaea and many Gram-negative bacteria; the systems found in other organisms and organelles are described elsewhere in this minireview series.

## Introduction

**Fig. 1:**
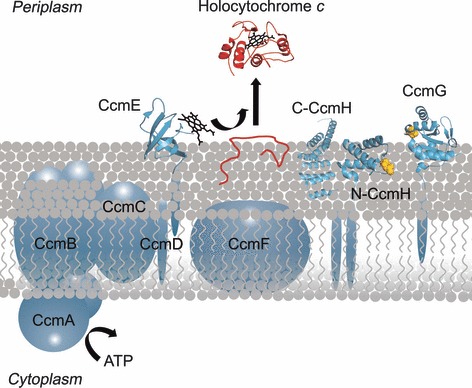
The cytochrome *c* maturation System I. The Ccm proteins (in blue) are all integral membrane proteins or are membrane-anchored with soluble domains in the periplasm (with the exception of CcmA, which hydrolyses ATP in the cytoplasm). The structures of the soluble domains of CcmE, CcmG and the N-terminal domain of CcmH have been solved and are shown on the periplasmic side (the Protein Data Bank accession numbers are 1LIZ [53], 2B1K [54] and 2HL7 [21], respectively). The structure of a paralog of the C-terminal domain of CcmH, NrfG, is also shown (Protein Data Bank accession number: 2E2E [23]). The holocytochrome *c* shown is from *Paracoccus denitrificans* (Protein Data Bank accession number: 155C). CcmH in *Escherichia coli* is a fusion of two proteins that occur separately in other organisms (CcmH and CcmI, labeled N-CcmH and C-CcmH). The apocytochrome *c* protein is shown in red, as is the holocytochrome *c* produced when heme (shown in black) becomes covalently attached. The cysteine residues, assumed to be involved in reducing the –CXXCH– motif in the apocytochrome, are shown in yellow.

**Fig. 2:**
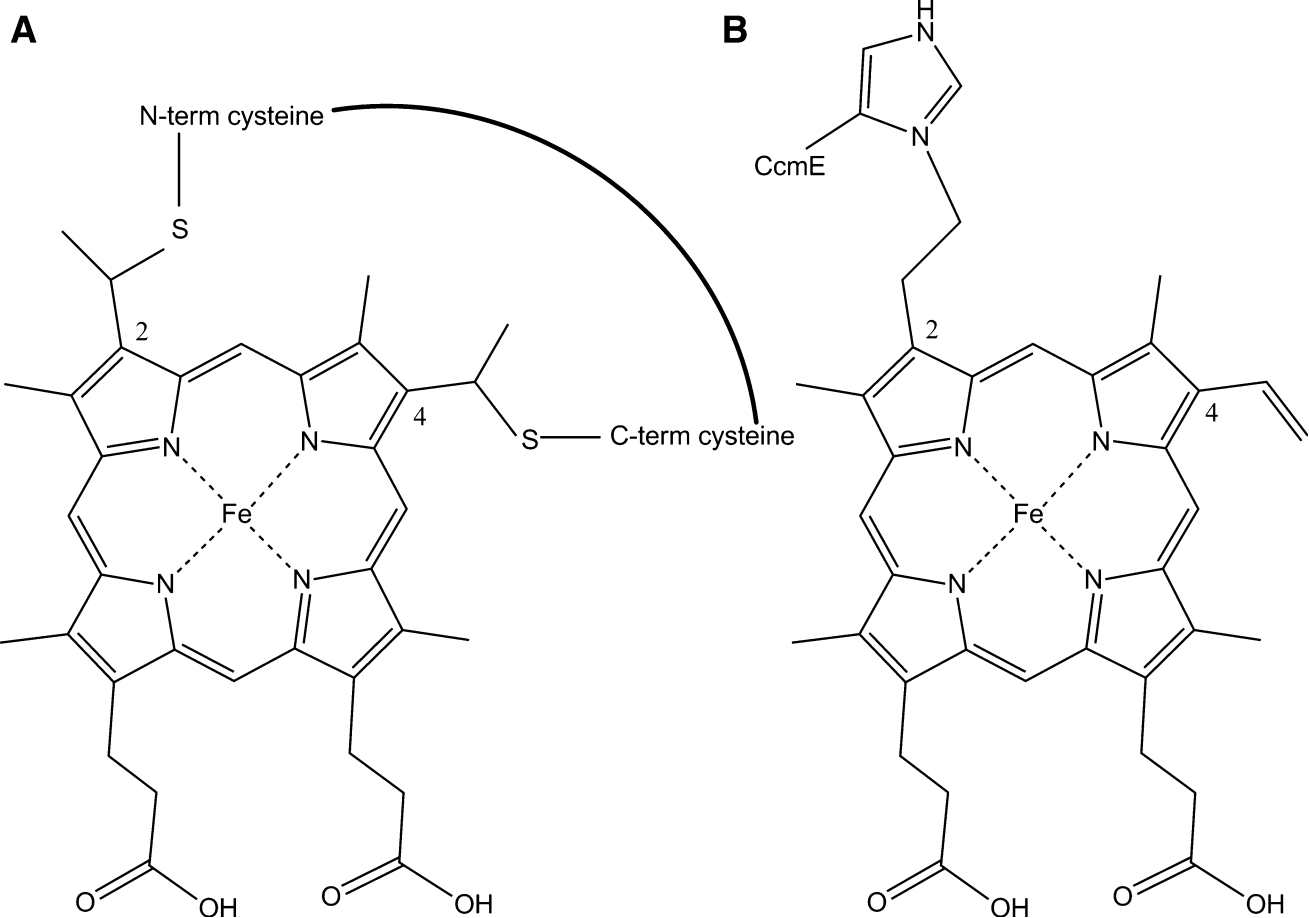
(A) Heme attachment to cytochrome *c* indicating the thioether bonds formed at the 2-vinyl and 4-vinyl heme positions to the two cysteine residues on the protein. The N′ and C′ orientations of the two cysteines are shown. (B) Heme attachment to the histidine side chain of CcmE [36].

The post-translational modification that covalently attaches heme to the –CXXCH– motif of cytochromes *c* is a protein-catalysed process, of which there are several types. System I is the most complex of the known systems and, in *Escherichia coli*, the eight cytochrome *c* maturation (Ccm) proteins (CcmABCDEFGH) (Fig. 1) are expressed from a single operon. It is notable that all the known biogenesis systems achieve the same stereochemistry and orientation of heme attachment to the protein; the N-terminal and C-terminal cysteines are always attached to the 2-vinyl and 4-vinyl groups of heme, respectively (Fig. 2A) [1,2]. The distribution and properties of the different biogenesis systems are reviewed elsewhere [3–5], as is the maturation of cytochromes *c* in mitochondria [6] and the formation of an unusual *c*-type cytochrome within the cytochrome *b* subunit of the cytochrome *b*_6_ *f* complex [7].

It is assumed that the formation of the cytochrome *c* thioether bonds requires reducing conditions, both to avoid formation of a disulfide bond within the –CXXCH– motif and to maintain the heme in the ferrous state. Amongst the evidence for this is that it is possible to incubate apocytochrome with ferrous heme *in vitro* and observe first the formation of a *b*-type cytochrome followed by the formation of the thioether bonds to give the *c*-type product [8]. A similar reaction occurs using zinc protoporphyrin IX, which can only access the +2 oxidation state [9]. Added to this is the unusual uncatalysed formation of thermophilic *c*-type cytochromes in the cytoplasm of *E. coli*, known to be a relatively reducing environment [10]. Although an *in vitro* chemical precedent can never be an absolute indicator of *in vivo* mechanism, we shall generally assume that the same reaction conditions are required *in vivo*. This is important because *c*-type cytochromes are formed in oxidizing parts of the cell, be it in the periplasm in Gram-negative bacteria, outside the cytoplasmic membrane of Gram-positive organisms or in the intermembrane space of mitochondria. *E. coli* and *Rhodobacter capsulatus* have featured in most recent studies of System I, along with experiments on the Ccm system found in plants. Below, we describe what is known from these studies about the transport of heme and apocytochrome, their handling in the periplasm, and their presentation to the Ccm proteins, in the context of *E. coli* as the model organism. The functions of the proteins are summarized in Table 1. It should be noted that, at present, conclusions drawn from experiments with *E. coli* cannot necessarily be extrapolated to *R. capsulatus* and *vice versa*. Another complementary review places more emphasis on *R. capsulatus* [11]. Finally, variations on the classical System I components are described (Table 1). These are of interest in their own right, and provide insight into the functions of the ‘standard’ Ccm proteins.

**Table 1:** Functions of the proteins (and variants) involved in cytochrome *c* biogenesis System I. All the Ccm proteins and DsbD are essential for cytochrome *c* maturation in *E. coli.* The involvement of DsbA has not been resolved. Note that, in *E. coli* and some other organisms, the CcmH protein contains two domains that are found as two separate proteins in many other organisms. Thus, the N-terminus of *E. coli* CcmH is known as CcmH elsewhere but the C-terminus of *E. coli* CcmH (C-CcmH) is known as CcmI when it occurs elsewhere. Although loss of the N-terminal region of *E. coli* CcmH results in loss of *c*-type cytochromes, the C-terminal domain is dispensable [26,28]. There are contradicting reports of the function and topology of CcmD [34,35]. The structures of DsbA and the soluble domains of CcmE, CcmG, N-CcmH and DsbD have been solved. The structures of the other proteins are unknown. –, no known variants.

Protein	Functions/features	Known variants
CcmA	Cytoplasmic ATP hydrolysis subunit of ABC protein that includes CcmB	–
CcmB	Membrane subunit of ABC protein involved in heme-handling by CcmE via an unknown mechanism	–
CcmC	Transmembrane protein providing heme to CcmE	–
CcmD	Small transmembrane protein that facilitates the interaction of other Ccm proteins	–
CcmE	Heme chaperone that binds heme covalently (to His in *E. coli*)	Covalent binding to Cys in System I^*^
CcmF	Large heme-containing transmembrane protein proposed to deliver reductant to heme on CcmE and to function in heme transfer from CcmE to apocytochrome	NfrE (required for biogenesis of NrfA nitrite reductase)
CcmG	Periplasmic thiol-oxidoreductase considered to be involved in the reduction of the cysteines in the -CXXCH- motif of apocytochromes	Absent, or equivalent protein not detected, in plants and System I^*^
N-CcmH	Periplasmic thiol-oxidoreductase considered to be involved in the reduction of the cysteines in the -CXXCH- motif of apocytochromes	NrfF (for biogenesis of NrfA nitrite reductase). Absent from System I^*^ in some cases
C-CcmH/CcmI	TPR-motif-containing protein considered to facilitate interaction with apocytochrome *c*	NrfG (for biogenesis of NrfA nitrite reductase)
DsbD	Transmembrane protein with two soluble periplasmic domains that transfers reductant from the cytoplasm to the periplasm	CcdA (a transmembrane reductant-transferring protein) found in some organisms
DsbA	Periplasmic thiol-oxidase functioning in disulfide bond formation	–

## Apocytochrome transport, handling and redox state control

Soluble periplasmic cytochromes *c* in Gram-negative bacteria have signal peptides that lead to their transport to the periplasm by the Sec system [12]; they are delivered across the membrane, N-terminus first, in their unfolded apoform. Membrane-associated cytochromes *c* also have their heme(s) attached by the periplasmic Ccm system. There is evidence that the two cysteines of a –CXXCH– motif can form a disulfide [8], as demonstrated by the need for a reducing protein (DsbD) in the oxidizing environment of the periplasm. The protein assumed to cause oxidation of the apocytochrome cysteines is DsbA, a powerful periplasmic oxidase that incorporates disulfides into extracytoplasmic proteins [13]. Some of the components of the bacterial Ccm system are generally considered (although not rigorously established) to return any disulfides to the two thiol state, which has to occur before heme attachment can proceed. One such component is the protein CcmG (Fig. 1), which is a periplasmic thioredoxin that is reduced by the transmembrane protein DsbD. DsbD is a unique three-domain protein that controls the redox state of the periplasm by transferring reductant from cytoplasmic thioredoxin via a thiol:disulfide cascade [7]. DsbD is essential for cytochrome *c* maturation and much progress has been made in understanding how it functions [14–16].

**Fig. 3:**
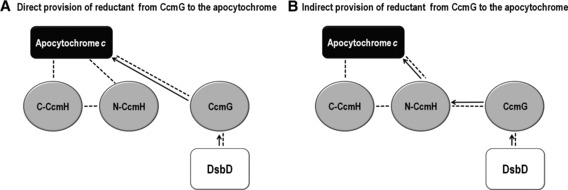
Possible interaction networks for CcmG, CcmH and apocytochrome *c* showing either direct (A) or indirect (B) provision of reductant from CcmG to apocytochrome *c*. The arrows indicate the possible pathways of reductant transfer and the dotted lines represent protein–protein interactions. (A) In the case of direct provision of reductant, CcmG would reduce the –CXXCH– motif in apocytochrome *c*. N-CcmH and C-CcmH would be involved in interactions with each other and with the apocytochrome *c* to facilitate heme attachment. (B) In the case of indirect provision of reductant, CcmG would reduce N-CcmH which, in turn, would reduce apocytochrome *c*.

It is not clear what receives reductant from CcmG; the possibilities are shown schematically in Fig. 3. It has been suggested that the environment of the redox-active pair of cysteines in CcmG is tuned to interact with the –CXXCH– motif of the apocytochrome [17]. Loss of CcmG in *E. coli* abolishes *c*-type cytochrome formation and this cannot be reversed by an exogenous reductant or by removal of the strongly oxidizing protein DsbA [18]. However, CcmG, in which one or both cysteines had been replaced by serine, allowed low level *c*-type cytochrome formation, which was enhanced to wild-type levels by thiol compounds [19]. It has also been suggested that, in *R. capsulatus*, CcmG lacking its thiols can interact with apocytochrome *in vitro* [20]. Thus, CcmG could interact directly with the apocytochrome *c* in a fashion that is not entirely dependent on the cysteines but is essential for the processing of the apoprotein *en route* to becoming a holocytochrome *c* (Fig. 3A). Alternatively, the pair of cysteines in the –CXXC– motif of the CcmH protein might be the redox partners of the apocytochrome *c* (Fig. 3B). Evidence for this includes *in vitro* binding studies [21] and a yeast two-hybrid study on mitochondrial CcmH [22]. An observation that the reduction of CcmG requires a functional CcmH [18] suggests that CcmH is the reductant for CcmG, in contrast to the usually accepted scheme.

CcmH in *E. coli* is a fusion of two proteins that are expressed separately in other organisms (CcmH and CcmI) (Fig. 1). The N-terminal domain of CcmH (N-CcmH in *E. coli*) is a membrane-anchored three helix bundle with a conserved pair of cysteines that is considered to function as a thiol-disulfide oxidoreductase [21]. CcmI (for which structural information can be inferred from the paralog NrfG [23]) is a tetratricopeptide repeat (TPR)-containing protein that has been proposed to interact with the apocytochrome protein. The majority of genera of proteobacteria have unfused CcmH and CcmI proteins. In six genera of γ-proteobacteria (including *Escherichia* and *Salmonella*), the majority of species have the proteins fused, although this arrangement is the exception overall. The cases where fusions are observed imply, at least, closely-related functions and the likely interaction of the two proteins.

Identification of the roles of CcmG and CcmH is further complicated by several sets of observations. First, it has been found that a variant of System I found in sulfate-reducing bacteria (called System I*; as described in more detail below) does not possess CcmH or CcmG and yet can function heterologously in *E. coli* lacking the endogenous Ccm system [24]. By contrast, the endogenous System I of *E. coli* will not function without CcmG or CcmH [25,26]. There is no obvious ortholog of CcmG in plant mitochondria [27], although this may be a failure of bioinformatic analysis to detect the gene. In summary, there is good evidence that CcmG tranfers reductant, although the function of CcmH/CcmI remains unclear. In *E. coli*, the CcmI part of CcmH is dispensable for *c*-type cytochrome production [26,28] but the same is not true for CcmI in *R. capsulatus* [11], whereas there is no evidence for a protein related to CcmI in the intermembrane space of mitochondria. At present, it appears that the TPR domains of CcmI ‘help’ in the formation of these cytochromes but, in some organisms, they are not required; it may emerge that the exact nature and expression level of the *c*-type cytochrome being investigated is an important consideration.

## Heme transport and delivery

Heme is synthesized in the bacterial cytoplasm and has to be delivered to CcmE on the other side of the membrane. There is no clear evidence as to how this occurs. The proteins CcmAB and C form a complex that is involved in delivering heme to the heme chaperone CcmE. Recent evidence suggests a role for CcmAB in heme release from CcmE [29,30]. The CcmA and CcmB proteins are members of the ATP-binding cassette family and initially were postulated to be heme transporters to the periplasm in bacteria. This role now appears to be very unlikely because heme still becomes attached to CcmE when the ATPase activity of CcmAB has been lost [29,30]. CcmC is required for heme attachment to CcmE and itself interacts with heme such that the latter is bound simultaneously by CcmC and CcmE [31]. It is tempting to assume that CcmC might also transfer heme across the membrane, although there is no evidence for this and, indeed, the lack of conserved histidines in transmembrane helices can be taken as evidence against this possibility [31]. Formation and breakage of the heme-CcmE bond has been demonstrated *in vitro* with purified apoprotein and heme [32], which suggests that the heme can either be coordinated by CcmC or by CcmE alone.

CcmE is a heme chaperone with a unique mode of covalent binding of the heme cofactor. It has a globular domain exposed to the periplasm and a membrane-anchoring N-terminal helix (Fig. 1). It has been known for some years that heme becomes covalently attached to a histidine of CcmE [33]. Replacement of this histidine with alanine not only prevents the covalent attachment, but also blocks *c*-type cytochrome synthesis. Evidence has been obtained showing that covalently bound heme on CcmE transfers *in vivo* to an apocytochrome *c* [33]. Remarkably, the heme on CcmE cannot be transferred onwards to an apocytochrome *c* if the ATPase activity of CcmA is lost [29,30]. Although CcmD is a small transmembrane protein, it has been shown to interact with CcmC and CcmE [34] and is required for the release of CcmE with heme bound from a complex including CcmC [35].

There are many uncertainties surrounding the CcmE protein. The covalent bond between heme and the histidine is sufficiently stable to withstand purification and the generation of a peptide from which the structure was determined [36]. The N-C bond involves the β-carbon of the vinyl group, which eventually becomes the methyl group of the heme in a *c*-type cytochrome (Fig. 2B). Thus, for the bound heme to be transferred directly to the apocytochrome, a hydride would have to leave the α-carbon atom in exchange for the attacking thiol. The leaving of the histidine side chain would have to be accompanied by capture of a hydrogen to generate the methyl group. Alternatively, as suggested by Kranz *et al.* [37], the histidine might depart from heme in the ferrous state via a reverse Michael reaction, thus transiently reforming the vinyl group and a noncovalently bound heme. Energy from ATP hydrolysis by CcmA might be needed to drive CcmC out of the heme coordination sphere and thus allow breakage of the heme to CcmE bond. An *in vitro* study showed that heme could transfer to an apocytochrome *c* provided that the CcmE carried a His-tag [32]. No such transfer was observed to a related apocytochrome *b* (i.e. lacking the heme-binding cysteines) suggesting, at least in the *in vitro* system, that ‘free’ heme was not formed by release from CcmE. Another puzzling aspect of CcmE is that other conserved residues around the histidine are dispensable but, if an extra alanine is inserted in this region, then heme attachment is abolished along with synthesis of holocytochrome *c* [38]. It could be that the interaction with a partner protein is compromised in this case. There are several lines of evidence showing tyrosine ligation of the heme iron in CcmE [39,40] but, unexpectedly, this side chain can be removed without loss of function [38]. Although the details of how CcmE functions remain to be elucidated, it clearly represents a key component of the Ccm system for providing the apocytochrome with heme.

## The heme attachment reaction

A variety of data indicate that System I recognizes only, or very little more than, the –CXXCH– motif of the apocytochrome. These include the observation that peptides with a –CXXCH– motif, but of only 12 amino acids can undergo heme attachment by the Ccm system [41], as well as the occurrence of close spacing of some –CXXCH– motifs observed in multiheme cytochromes. There is evidence that CcmG and CcmH can interact with CcmF (Fig. 1), which appears to be at the heart of attaching heme to cytochromes. CcmF interacts with CcmE [42] and with CcmH [43]. CcmH has been shown to interact with apocytochrome [22] in a plant system and thus a situation can be envisaged in which CcmH binds the apocytochrome and then presents it to CcmF for heme attachment, with heme provided by CcmE. Conserved residues in the periplasmic loops of CcmF, especially tryptophans, are envisaged to be involved in handling heme as it is incorporated into the apocytochrome [43]. It has long been a puzzle as to why CcmF has so many (> 11) transmembrane helices, which might suggest a role as a transport protein. Important recent work has detected heme bound to CcmF [44] and its suggested role is to supply reductant to maintain the heme attached to CcmE, which is destined to be transferred to an apocytochrome, in the ferrous state. CcmF in plants is made up of three separate polypeptide chains and the function of the protein can be envisaged as being split between these three segments. The detection of an additional sulfur atom between the heme-binding cysteine and the heme in the holocytochrome product when the spacing between the heme-binding cysteines is altered [45] provides a rare clue that heme attachment might involve some kind of activation reaction involving a third species other than heme and apocytochrome, and that the reaction ‘goes wrong’ when the spacing is nonphysiological. The nature of the putative third component is unknown.

## Variations of System I

### System I*

Both the formation of the covalent bond between heme and a histidine in CcmE and also its subsequent breakage to allow heme to attach to an apocytochrome clearly represent novel biochemical reactions. It is therefore remarkable that analysis of genomic data revealed that the assumed central features of the Ccm system might not be essential [46]. These features were the presence of the essential heme-binding histidine in CcmE, and the protein CcmH, which is required for cytochrome *c* production in *E. coli.* It was observed that the CcmE proteins found in archaea all had a cysteine in place of the heme-binding histidine seen in previously studied Ccm systems. The archaeal genomes also lacked a gene for CcmH. Some bacteria, including *Desulfovibrio* species, also contained genes for the variant Ccm system, called System I*. The necessity for the missing components was determined experimentally by testing whether the Ccm operon from *Desulfovibrio* could produce holocytochromes *c* in *E. coli* [24]. Holocytochrome *c* was produced despite the absence of CcmH and CcmG, and with a cysteine-containing CcmE, which was shown to bind heme covalently. If the binding is analogous to that of heme to the histidine of ‘standard’ CcmE, then the bond between heme and CcmE* will be a thioether bond. The ability of the proteins to accommodate cysteine-based heme chemistry, instead of the unusual histidine-based heme handling, was unexpected. Thioether bonds are usually very stable, as for example in *c*-type cytochromes. Thus, it is not obvious that such a bond between heme and a cysteine of CcmE could be easily broken by a reverse Michael reaction.

Analysis of the larger number of genomes now available has revealed many more examples of CcmE proteins containing cysteine in place of the heme-binding histidine. δ-Proteobacteria make up the largest proportion; chloroflexi and firmicutes also feature. The majority of the variant CcmEs have a CPSKY motif (25 of 36 System I*-containing bacterial genomes examined); only four examples were identified in which the Y was absent and replaced with an F. Of bacteria with the System I* CcmE, approximately one-third of them contain CcmH, and so the absence of CcmH is not a characteristic feature of this variant system. Nevertheless, at first glance, work with System I* suggests that CcmH is not absolutely needed to work alongside CcmF to produce *c*-type cytochromes. In System I*, CcmI appears to enhance the production of *c*-type cytochromes but is not absolutely required [24].

### The Nrf proteins

The bacterial nitrite reductase NrfA is a periplasmic pentaheme cytochrome *c* in which one of the heme attachment motifs is an unusual –CXXCK–, which forms the active site of the enzyme. Three proteins from the Nrf operon, NrfEFG, have been implicated in attaching heme to this motif [47] (Table 1), although it has been shown that NrfF is not essential [48]. NrfF is a paralog of N-CcmH, which is essential for heme attachment to a –CXXCH–. It is not clear why the Nrf biogenesis system would not require this protein but, because the Ccm proteins attach heme to the four –CXXCH– motifs in NrfA in the absence of NrfEFG [49], it could be that the conformation of the substrate apocytochrome recognized by the Nrf proteins is different from that normally recognized by the Ccm proteins. It is possible that the –CXXCK– is less prone to disulfide bond formation. The structure of NrfG has indicated a potential binding site for NrfA, via the TPR motifs in NrfG [23]. TPR motifs are widely involved in facilitating protein–protein interactions. If it is the case that CcmF plays a core role in attaching heme to –CXXCH–, then it is likely that the paralog NrfE has the equivalent role when a –CXXCK– sequence is being handled.

Examination of the frequency and arrangement of the Nrf genes in the many bacterial genomes now available shows that 55 unique species had a gene for a NrfA (occasionally containing seven –CXXCH– motifs rather than four, in addition to the –CXXCK–). The gene arrangements show that, in 18 of these, the NrfA gene is in an operon with NrfEFG. We note that NrfF and NrfG are not found together as frequently as NrfE and NrfF; this is unanticipated because the function of NrfF and NrfG is expected to be closely related, not least because the Ccm paralogs (CcmH and CcmI) are fused proteins in *E. coli*.

In an organism that makes NrfA using cytochrome *c* biogenesis System II (described elsewhere in this minireview series [5]), a variant protein of that system is also required for heme attachment to the –CXXCK–, namely NrfI [50]. We also note that a CX_15_CH heme-binding motif in a *Wolinella* cytochrome *c* has been identified and a dedicated biogenesis protein is also required for heme attachment to this unusual motif [51].

## Conclusions

Much has been learned about the Ccm apparatus, although many aspects remain to be unravelled, and the recent discovery of System I* raises new and unexpected questions. Prominent amongst those concerns the interaction of heme with CcmE. The idea of a unique type of bond between heme and the side chain of an absolutely essential histidine in CcmE has been well developed over the last 10 years. If this is so important for the function of CcmE, then it is unexpected that this essential histidine is replaced by an cysteine in some Ccm systems. Neither system can tolerate a switching of amino acid at this position (i.e. histidine to cysteine or *vice versa*). Does this mean that there is an important difference in mechanism for System I as opposed to I*? CcmH is often seen alongside CcmF as being essential for interaction with apocytochromes *c* in the heme attachment process, although some, but not all, Systems I* can function without this protein. This could mean that System I* has compensatory features in other Ccm proteins, although bioinformatic approaches have provided no evidence for this. However, based on information from all experimental systems [11,37], it might be argued that the fundamental core of the Ccm system is the proteins ABCDEF. It is possible, as suggested from work on *R. capsulatus*, and seen analogously with System II, that CcmG is required to reverse the formation of disulfides by the Dsb system. However, as discussed previously [11,20], CcmG may have a key role other than in the reduction of the disulfide in an apocytochrome *c*. There are clearly many key issues that need to be studied to allow an understanding of this post-translational system whose function is essential for many eukaryotic organisms [3] and for many modes of respiration, from aerobic to many types of anaerobic respiration including sulfate, for which the involvement of *c*-type cytochromes came as a big surprise almost 60 years ago [52].

## Abbreviations

Ccm: cytochrome *c* maturation
TPR: tetratricopeptide repeat
